# Mental Health Responses to Covid and Lock-down in Auckland, New Zealand

**DOI:** 10.1192/j.eurpsy.2023.870

**Published:** 2023-07-19

**Authors:** I. Soosay

**Affiliations:** Mental Health & Addictions, Te Whatu Ora / Health New Zealand Counties Manukau, Auckland, New Zealand

## Abstract

**Introduction:**

The Government of New Zealand closed the borders and introduced a number of restrictions following the first reported case of Covid-19 in February 2020. Comprehensive measures to control the outbreak included a strict managed isolation and quarantine (MIQ) system and rolling lockdowns that restricted movement. There were concerns about the mental health impact on the population. This presentation will outline the approach to maintaining social cohesion and supporting the psycho-social needs of the population through the pandemic.

**Objectives:**

An overview of the Covid-19 response will be presented, including the Covid-19 alert systems and the strategies to support particular populations.

**Methods:**

This will include:

1. The mental health support for the 229,000 people subjected to managed isolation and quarantine in government facilities

2. Psycho-social interventions for our Maori and Pacific populations

3. Specific approaches for people with severe mental disorders, including vaccination strategies and supporting people in the community with Covid-19

4. Interventions for homeless and socially vulnerable populations

**Results:**

Reflections and learnings from our apprach will be shared.

**Conclusions:**

The government ended the Covid-19 Framework in September 2022 and retrurned to fully open borders in October 2022. There have been over 1.8 million cases and 2000 deaths during the period of restrictions.

**Disclosure of Interest:**

None Declared

